# The Role of Whole-Gland and Focal Cryotherapy in Recurrent Prostate Cancer

**DOI:** 10.3390/cancers16183225

**Published:** 2024-09-22

**Authors:** Faozia Pio, Andeulazia Murdock, Renee E. Fuller, Michael J. Whalen

**Affiliations:** Department of Urology, George Washington University School of Medicine, 2300 I St NW, Washington, DC 20052, USA

**Keywords:** prostate cancer, salvage cryotherapy, salvage therapy, radiation therapy failure, biochemical recurrence, cryoablation, salvage HIFU

## Abstract

**Simple Summary:**

The majority of newly diagnosed prostate cancer patients are eligible for active surveillance, and those at moderate to high risk frequently receive definitive treatments such as radiotherapy or radical prostatectomy. A considerable proportion of patients will experience biochemical recurrence, as well as localized and distant metastatic recurrence following definitive treatment. Salvage cryotherapy has emerged as a safe and effective alternative for treating localized recurrence. This review examines the oncologic and functional outcomes of whole-gland and focal salvage cryotherapy, as well as the crucial role of multiparametric prostate MRI and PSMA-targeted PET imaging. The outcomes of cryotherapy compared to those of other salvage ablation techniques like high-intensity focused ultrasound (HIFU) are also explored.

**Abstract:**

Prostate cancer is the most common non-cutaneous malignancy in men, with the majority of newly diagnosed patients eligible for active surveillance. Despite definitive treatment, a considerable percentage of men will experience biochemical recurrence and even regional and distant metastatic recurrence after radiation therapy or radical prostatectomy. Salvage prostatectomy, while oncologically effective, poses significant morbidity with poor functional outcomes. Salvage cryotherapy has emerged as a promising alternative for localized recurrence, demonstrating safety and efficacy. This review examines the oncologic and functional outcomes of whole-gland and focal salvage cryotherapy, including disease-free survival, cancer-specific survival, and overall survival. The crucial role of multiparametric prostate MRI and evolving role of next-generation PSMA-targeted PET imaging are also examined. The comparison of outcomes of cryotherapy to other salvage ablation modalities, such as high-intensity focused ultrasound (HIFU), is also explored.

## 1. Introduction

Prostate cancer is the most common non-cutaneous malignancy in men and the second-leading cause of cancer death in American men, behind only lung cancer. According to the American Cancer Society’s projections, about 299,010 new cases will be diagnosed in the United States in 2024 [1]. While active surveillance is common as a primary strategy for low-risk patients, those with intermediate to high risk often undergo definitive treatments such as radiotherapy or radical prostatectomy [2]. Unfortunately, about 32% of patients who undergo radiation therapy [3] and more than 40% of men with intermediate or high-risk prostate cancer who undergo radical prostatectomy will experience a biochemical recurrence [4]. Effective treatment of local recurrence of prostate cancer following radiotherapy remains a challenge. A high rate of oncologic control is achieved with salvage prostatectomy; however, this procedure has been considered relatively morbid, with a high rate of incontinence and other morbidities [5]. 

Cryotherapy has been shown to be safe in the setting of localized prostate cancer recurrence. For many patients seeking a minimally invasive treatment for prostate cancer recurrence following prior radiation therapy, salvage cryotherapy may be a helpful option in reducing morbidity, including erectile dysfunction and urinary incontinence [3]. This review examines clinical outcomes of whole-gland and focal salvage cryotherapy for recurrent prostate cancer, including disease-free survival, cancer-specific survival, and overall survival. The critical role of multiparametric prostate MRI (mpMRI) and growing role of next-generation PSMA-targeted PET imaging are also examined. The comparison of outcomes of cryotherapy to other salvage ablation modalities, such as high-intensity focused ultrasound (HIFU), is also explored. 

This narrative review was conducted primarily through use of the PubMed database. Given the longitudinal reporting nature of this review, there were no limits applied to the publishing date or languages of published studies. Search terms used included “cryotherapy”, “cryosurgery”, and “cryoablation”.

## 2. Cryoablation Technique

Cryoablation, also called cryotherapy or cryosurgery, is categorized as focal therapy, or a treatment targeting a specific region of the prostate. Initially performed as an open perineal procedure for clinically significant prostate cancer in the 1960s, it frequently resulted in high morbidity. In 1993, cryoablation was reintroduced, and real-time monitoring of the process was achieved using transrectal ultrasound (TRUS) [6]. One of the unique features of image-guided focal cryoablation is that it uses multiparametric prostate MRI (mpMRI) to precisely localize the site of clinically significant cancer, as determined by MRI-TRUS prostate fusion biopsy. The mpMRI is also able to show gland size, regions of carcinoma, and their topographical location in relation to other important structures, including the bladder neck, urethra, rectum, and neurovascular bundle. 

**Figure 1:**
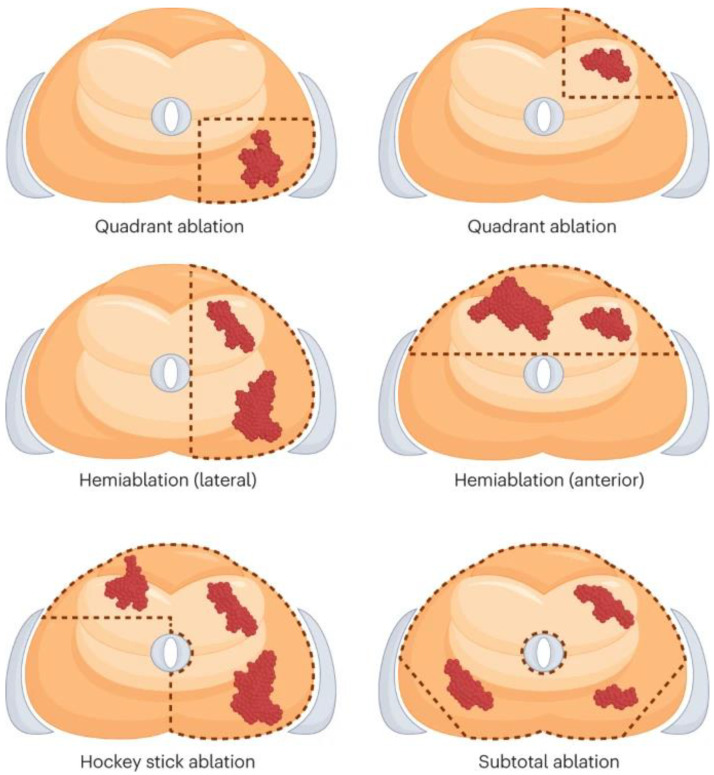
Common ablation technique patterns [7]. Reprinted with permission from ref. [7]. Copyright 2023 Elsevier.

Cryoablation needles are then percutaneously inserted through the perineum into the target locations using a TRUS ultrasound probe and brachytherapy template grid to form “ice balls”, creating an aggregate ablation zone. Freehand approaches without the brachytherapy template grid have also been described. The standard of treatment involves two freeze–thaw cycles to optimize tissue damage. Urethral sloughing is avoided with the use of a urethral warming catheter. Depending on the location of the cancer, the ablation configuration can be customized or may employ the quadrant, hemi-, hockey stick, or sub-total ablation patterns. The diagram below provides a description of these configurations (Figure 1).

Supercooled gas flows through unique hollow needles called cryoprobes. The prostate can undergo freezing through a fairly controlled mechanism when cryoprobes are used with thermocouples—needles that detect tissue temperature at crucial surrounding structures, such as the cavernous nerves and the external urinary sphincter. Modern techniques that make use of MRI-TRUS fusion software can also be used to guarantee precise and sufficient coverage of the index lesion. Such commercially available MRI-TRUS fusion devices include Urostation, which uses a 3D ultrasound probe to overlay images [8]. Artemis and BioJet overlay images using robotic tracking by a mechanical arm with encoders built in. Images are overlaid by the UroNav device via electromagnetic tracking. 

**Figure 2:**
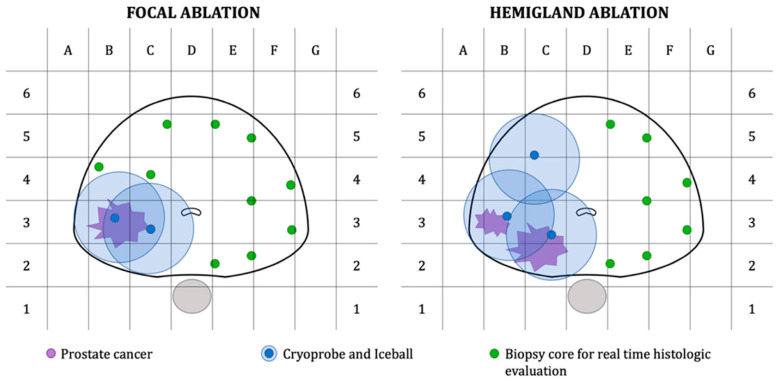
Graph representation of treatment plan and biopsy core template for patients undergoing focal or hemigland cryoablation [14], reprinted with permission from ref. [14]. Copyright 2021 *Cancers*. Licensed under CC BY 4.0.

Time required for image alignment has been found to vary, with one study by Valerio et al. requiring 12–15 min. However, the utilization of MRI-TRUS facilitated the placement of probes to create treatment zone margins [9]. The suitable treatment margin is controversial. It requires adjustments for radiolucent tumor extension beyond the perceived border of the visible ROI, patient motion, anatomical distortion from needle placement, and registration error from the fusion platform technology. Whole-mount pathologic sections have suggested up to 1 cm extension beyond the radiographic border of the tumor [10]. To ensure that no malignant tissue is left behind, the consensus among experts is that the ablation zone should encompass approximately 1 cm of the surrounding tissue around the tumor [11,12,13]. In order to guarantee sufficient coverage of the cryoablation area, the positioning should adhere to the triangle pattern depicted below (Figure 2).

Patients receive postoperative care, similar to that of other transurethral procedures, including the use of a catheter for several days following the procedure, and appropriate pain control. For men with large projected ablation zones, preemptive suprapubic tube installation may be considered just prior to the cryoablation in the same setting. Prophylactic antibiotics, alpha antagonists, and analgesics are added as needed.

## 3. Cryotherapy in Various Salvage Settings

For many patients seeking minimally invasive curative salvage treatment for prostate cancer recurrence post-radiation therapy, salvage cryotherapy may be an effective option, and may obviate the need for androgen deprivation therapy with its consequent undesirable side effects (i.e., male menopause syndrome and metabolic syndrome). Savafy et al. (2019) conducted a retrospective review of *n* = 75 men who underwent salvage cryoablation for biopsy-proven recurrence post-radiation therapy. Of this group, *n* = 6 underwent radiation therapy with androgen deprivation therapy. Seventy received whole-gland cryotherapy and five underwent hemiablation. They found that a post-cryotherapy PSA nadir of ≤0.5 ng/mL was associated with a biochemical progression-free survival (BPFS) of 79.7% at 3 years and 64.7% at 5 years [5]. Post-cryotherapy PSA nadir > 0.5 ng/mL was associated with a BPFS of 5.6% at 3 years and 0% at 5 years (*p* < 0.0001) [5]. It was concluded that PSA after salvage was the strongest predictor of BPFS. 

In a systematic review, Chin and Lynn (2022) assessed post-procedural complications of salvage cryotherapy and found that five studies reported erectile dysfunction ranges between 25.0 and 86.2% and urinary retention ranges between 2.13 and 25.3%. In four studies, rectourethral fistulae ranged from 1.27 to 3.7%, and in two studies, pelvic perineal pain ranged from 10.71 to 31.25% [15]. 

It should also be noted that the criterion for success of cryotherapy, either primary or salvage, is adapted from radiotherapy metrics, namely the Phoenix criteria or a PSA rise of 2 ng/mL above the nadir value. This metric reflects the fact that radiotherapy is not an ablative (i.e., tissue-destroying) modality, but damages prostate cancer cell DNA and then prompts these cells to undergo apoptosis later. Furthermore, some periurethral prostate tissue is excluded from the highest radiation fields to prevent urethral stricture/sloughing. Although the Phoenix criteria have been applied to cryotherapy, this may not be apt given the fact that cryo is a tissue-destroying ablative modality. However, as the PSA nadir depends on the volume of prostate tissue ablated, and similarly periurethral tissue is not fully ablated, the post-treatment nadir is variable among patients after whole-gland cryoablation. Even more variable is the PSA decline after focal cryoablation. Therefore, although PSA nadir is associated with treatment success (i.e., biochemical recurrence-free survival; see below) and despite its considerable limitations, the Phoenix criteria have been adopted as the convention for cryoablation efficacy (especially in retrospective studies), given the lack of other reliable metrics. Certainly, other parameters such as post-ablation multiparametric MRI and even post-ablation routine biopsy should be strongly considered given the limitations in biochemical monitoring with PSA.

To put the success rates of salvage cryotherapy in the context of outcomes from alternative salvage treatments, systemic androgen deprivation therapy (ADT) is often used. A 2008 study that used the Cancer of the Prostate Strategic Urological Research Endeavor (CaPSURE) database analyzed *n* = 1590 men (30%) who experienced disease recurrence out of a total of 5277 men with prostate cancer [16]. A total of *n* = 1003 men underwent radical prostatectomy (RP), and *n* = 587 men received ERBT as their initial treatment. Androgen deprivation therapy (ADT) was the most common salvage treatment in both groups. The study did not report whether continuous or intermittent ADT was administered. At a mean of 43.6 months and 43.8 months, respectively, salvage therapy was not successful for 420 men (68%) in the RP group and 319 men (74%) in the EBRT group (*p* = 0.95) [16]. Compared to the 311 men who did not fail salvage therapy, they had a higher overall death rate (24.8% vs. 6.9%, respectively; *p* < 0.001) [16]. It must be noted that the direct comparison of salvage cryotherapy outcomes to this particular study does not account for the fact that many patients undergoing salvage ADT do so due to more advanced disease, which may exclude them from salvage local therapy. Therefore, outcomes would be expected to be poorer for the salvage ADT group due to this inherent selection bias. 

### 3.1. Post-Radiation Therapy Recurrence

Although there are multiple treatment alternatives for salvage therapy, many patients with cancer recurrence following radiation therapy receive systemic androgen deprivation therapy (ADT) without salvage local therapy. Furthermore, men with slow PSA doubling time may even best be served by watchful waiting, given the low prostate cancer-specific mortality rate in this setting. 

The modern era has witnessed a revolution in radiographic staging in the setting of biochemical recurrence. Given the limited sensitivity of conventional imaging (i.e., CT scan and nuclear medicine Tm99 bone scan) to localize the site of recurrence, the efficacy of salvage therapy was often prognosticated based on the original disease pathology, pathology at recurrence, and PSA level prior to salvage treatment, with thresholds of 5 ng/mL and 10 ng/mL proposed (see Whole-gland salvage cryotherapy below). Contemporary staging involves use of prostate-specific membrane antigen positron emission tomography (PSMA PET) to enhance sensitivity of recurrent disease detection and most importantly localization (i.e., prostate gland, regional pelvic lymph nodes, and distant metastatic sites). Certainly, any local salvage treatment success is predicated upon localized, rather than regional or metastatic, disease recurrence. The predictive capacity of dual data points of the PSA threshold and PSMA PET findings warrant further research. Also, many of the studies cited below are in the pre-PSMA PET era and thus this realm will certainly evolve.

- Radiographic Staging at Recurrence
  (i) PSMA PET

Recent research suggests that PSMA PET, as opposed to conventional imaging, has higher detection rates in patients with biochemically recurrent prostate cancer following radiation therapy [17,18,19]. Furthermore, a strong correlation has been found between rising PSA levels and the detection of recurrence on PSMA PET scans. Perera et al. (2020) performed a systematic review and meta-analysis of the utility of ^68^Ga-PSMA-PET in assessing advanced or biochemically recurrent prostate cancer. In their analysis, which included six studies providing data following primary radiotherapy failure, they discovered that the percentage positivity of PSMA-PET scans for assessing recurrence increased with rising pre-PET PSA levels [20].

A 2017 study conducted at Royal North Shore Hospital in Australia investigated the role of ^68^Ga-PSMA-PET in patients with post-external beam radiotherapy treatment (EBRT) biochemical failure, defined as nadir PSA ≥ 2 ng/mL [17]. Patient selection included a cohort of *n* = 419 men treated with image-guided EBRT at a dose of 78 or 82 Gy +/− ADT between 2007 and 2014. After a median follow-up of 50 months, *n* = 70 men (17%) had post-EBRT biochemical failure, 13 of whom had died. Of the *n* = 57 surviving patients with biochemical recurrence, 5 had metastases detected on a CT or bone scan, and 48 underwent PSMA scanning within a median of 3 months (range: 1–57 months) [17]. All patients were scanned from the skull vertex to knees for a minimum of 60 min following injection. Increased uptake within the prostate or seminal vesicles (standardized uptake value, or SUV max > 3.3) was used to define local recurrence. Distant disease included lymph node, bone, or visceral metastases. 

In all cases, the PSMA was unequivocally positive. The site of failure following dose-escalated EBRT was mostly distant, 25 (52%) outside the prostate: 5 in bones, 16 in lymph nodes, 3 in both bones and lymph nodes, and 1 in the lungs. A total of 15 (31%) had failure within the prostate and in either lymph nodes (11), bones (3), or both (1), and 8 (17%) had isolated local recurrences [17]. Patients treated with high-dose (>80 Gy) compared to those treated with low-dose ERBT (≤80 Gy) had significantly higher odds of distant failure as opposed to local failure (OR: 34.5, 95% CI: 3.99–297.99). All things considered, Gleason scores ≥ 8 (17% vs. 9%, *p* = 0.011) and initial PSA > 1 0 ng/mL (16% vs. 8%, *p* = 0.04) were the only predictive factors for biochemical failure [17].

A similar retrospective study of *n* = 118 men with biochemical recurrent prostate cancer (nadir PSA ≥ 2 ng/mL) was conducted by Einspieler et al. [18]. Participants included 77 men who underwent EBRT and 41 who underwent brachytherapy as primary treatment. ADT was administered to 45 patients at least six months prior to ^68^Ga-PSMA-PET. Recurrence detection rates were stratified by PSA: 36/44 (81.8%) for PSA of 2 to <5 ng/mL, 41/43 (95.3%) for PSA of 5–<10 ng/mL, and 30/31 (96.8%) for PSA ≥ 10 ng/mL (*p* = 0.038) [18]. PSMA showed pathologic findings suggestive of recurrence in 107/118 (90.7%) men. Detection rates were significantly higher in patients after EBRT (75/77, 97.4%) compared to after brachytherapy (32/41, 78%) (*p* = 0.340). Local recurrence was detected in 68/107 (63.5%), distant lesions in 64/107 (59.8%), and both in 25/107 (23.4%) [18]. Interestingly, the detection rate was significantly higher in those who received ADT (97.7% vs. 86.3%, *p* = 0.0381), but this was independent from a primary Gleason score ≥ 8 (92.0%) versus ≤7 (90.2%, *p* = 0.6346). The SUV max and SUV mean were significantly associated with PSA and concomitant ADT (SUV_max_: *p* = 0.018 and 0.004; SUV_mean_: *p* = 0.025 and 0.007, respectively) [18].

Between November 2018 and August 2019, *n* = 208 patients with biochemical recurrent prostate cancer (nadir PSA > 2 ng/mL) were enrolled in the 2021 CONDOR phase 3 prospective multi-center study to evaluate the diagnostic performance of PMSA-based ^18^F-DCFPyL PET/CT [19]. Of all participants, 139 (68.8%) had PSA levels < 2 ng/mL and 31 (14.9%) had radiation therapy as their primary treatment. The recurrence detection rate was defined as the percentage of positive PMSA-based scans found by three independent, blinded central imaging readers. Based on a composite standard of truth (SOT), all patients with positive PMSA-based scan results were scheduled for additional exams to confirm any suspected lesions. The study used the correct localization rate (CLR), a novel endpoint recommended by the FDA, to measure the positive predictive value (PPV). CLR was defined as the percentage of patients with a one-to-one correspondence between at least one lesion identified on PMSA-based scans and the composite SOT.

The results showed that the detection rate rose with increasing PSA levels ranging from 36.2% (<0.5 ng/mL) to 96.7% (≥5 ng/mL) [19]. At least one lesion was detected in 59.1–65.9% of patients; CLR ranged from 84.8 to 87.0%; and PPV was consistently high across all anatomic regions. The PPV in the prostatic region ranged between 75.0% and 83.3% among the three readers [19]. Similarly, the PPV was between 67.2% and 72.7% for pelvic lymph nodes and ranged from 67.3 to 69.8% for the extra-pelvic regions [19]. It is important to keep in mind that primary treatment groups were not taken into account when analyzing the results. The findings of the PSMA studies are summarized in Table 1 below. Although dependent upon the pre-salvage treatment PSA and Gleason score, given that only 16.7–40.2% of patients have prostate-only disease recurrence, this information is crucial for optimal patient selection for salvage prostate-directed cryotherapy. The modern era should certainly witness improvements in salvage treatment efficacy when informed by more accurate PSMA PET staging.

  (ii) Multiparametric MRI

Following initial therapy, mpMRI can be used to visualize normal treatment changes and residual disease. It can also be used to diagnose locally recurrent disease. The multiparametric method combines anatomic sequences [T1-weighted (T1W) and T2-weighted MRI (T2W)] with functional imaging sequences, including diffusion-weighted imaging (DWI) and dynamic contrast-enhanced imaging (DCE) [21,22]. The mpMRI offers the most information for locating and identifying clinically significant disease. Irradiated prostatic tissue loses its normal anatomy, and PSA nadir is not reached as quickly as post-radical prostatectomy. Structures surrounding the prostate appear different as well, compared to their pre-treatment appearance. When using mpMRI, recurrent disease appears as a nodular lesion that is hypointense relative to normal prostatic tissue on T2W MRI [21]. DCE MRI provides information about vascularity, permeability, and contrast uptake. Together, nodular recurrence may demonstrate growth relative to the atrophic gland and neovascularization [21,23]. On DW MRI, recurrent lesions have low-apparent-diffusion-coefficient (ADC) maps and hypersensitivity. Nonetheless, it is possible to observe false positives on ADC due to hemorrhage, dysplasia, and high-grade prostatic intraepithelial neoplasia [21,24].

In a study analyzing the use of DWI and T2W for predicting locally recurrent prostate cancer in *n* = 36 men following EBRT, Kim et al. (2009) found that combined DWI and T2W showed greater sensitivity compared to T2W alone (62% vs. 25%, *p* < 0.001) [25]. There was no significant difference in the specificity of the combined imaging vs. T2W alone (97% vs. 92%, *p* > 0.05). Additionally, the area under the curve (AUC) of the Receiver Operating Characteristic (ROC) curve during an accuracy analysis of the combined imaging was significantly higher than that of T2W alone (87.9% vs. 61.2%, *p* < 0.01). For DWI + T2W and T2W alone, the PPV was 91% and 57%, and the negative predictive value (NPV) was 81% and 74%, respectively [25]. In another study of *n* = 24 men with rising PSA levels following ERBT, Kim et al. (2010) found that the sensitivity and specificity of DWI (49% and 93%), DCE (49% and 92%), and combined DWI and DCE (59% and 91%) were higher than T2W alone (27% and 80%) (*p* < 0.008) [26]. TRUS-guided biopsy was performed if recurrence was suspected in the prostate as seen at MRI. AUC of DWI and DCE (86.3%) was significantly higher than that of T2W (59.4%), DCE (73.7%), and DWI (78.2%) alone [26].

Studies on the efficacy of mpMRI in identifying recurrent disease after brachytherapy are scarce. This may be the result of the majority of brachytherapy patients having very low-risk primary disease. One study analyzed the use of MRI for detecting locally recurrent prostate cancer in *n* = 16 men with biochemical failure following high-dose-rate brachytherapy. The sensitivity and specificity for T2W were 27% and 99%, respectively; for DCE, they were 50% and 98%; and for DWI, they were 68% and 95% [27]. They found that mpMRI achieved the highest sensitivity (77%) but with slightly lower specificity (92%) [27]. The MRI findings are summarized in Table 2 below.

  (iii) Combining PSMA PET and Multiparametric MRI

In a study by Radzina et al., it was found that mpMRI had superior results compared to PSMA PET in the detection of local prostate cancer recurrence with sensitivity, specificity, PPV, and NPV values of 90.9%, 94.7%, 90.9%, and 94.7% vs. 94.7%, 63.6%, 77.8%, 58.3%, and 77.8%, respectively. For lymph node metastases, PSMA PET was superior to mpMRI with a sensitivity, specificity, PPV, and NPV of 83.3%, 80%, 80%, and 100% vs. 41.7%, 94.4%, 83.3%, and 70.8%, respectively [28]. Although an NPV of 100% was reported, it should be noted that the study was limited by its small patient size as well as an even smaller number of patients who underwent histopathological assessment of lesions seen on PSMA PET and/or MRI. Other studies contrast with this value, reporting NPV numbers as low as 41% [29,30]. In addition, it is important to include how this study defined ‘true positives’. Radzina et al. determined the reference standard based on the opinions from multidisciplinary team discussions that included nuclear medicine physicians, radiologists, and urologists [28].

Another study by Jannusch et al. examined the respective strengths of MRI and PSMA PET components in a combined MRI/PSMA PET imaging modality. It was found that the combination of both improved the tumor localization in patients with prostate cancer recurrence. More specifically, the study found that MRI detected local recurrence more accurately in comparison to PSMA PET (100% vs. 93% in patients with a PSA of <1.69 ng/mL, 100% vs. 87% in patients with a PSA > 1.69 ng/mL) [31]. However, PSMA PET detected distant lymph node metastases more accurately (93% vs. 90% in patients with a PSA of <1.69 ng/mL, 96% vs. 87% in patients with a PSA > 1.69 ng/mL) [31]. Authors in the study also defined a reference standard through a consensus reading by experienced imaging readers as well as information available from previous studies on PET/MRI. These findings are summarized in Table 3. 

A third study by Albisinni et al. performed a systematic review of novel imaging techniques in the context of recurrent and metastatic prostate cancer. It was found that mpMRI performed well in local detection recurrences with sensitivity rates as high as 98% and diagnostic accuracy as high as 93%. PSMA PET on the other hand performed well in detecting both local and distant recurrences with a sensitivity as high as 98% [32].

In a prospective study, Rasing et al. (2022) determined the PPV of combining PSMA PET and mpMRI and assessed whether pathology verification with MR-targeted biopsies was still necessary for patients with recurrent prostate cancer. A total of *n* = 41 patients with suspected local recurrence post-radiation therapy were imaged with ^68^Ga-PSMA-PET and mpMRI and subsequently had MR-guided targeted biopsies performed. They found that 40 (97.6%) patients had positive biopsies; hence, combined imaging with PSMA PET and mpMRI had a PPV of 97.6% for the detection of a local recurrence [33]. All patients had concordance in tumor locations within the prostate and/or seminal vesicle, and two patients had a unifocal lesion on one imaging modality when the other modality revealed bilateral involvement. They concluded that biopsy can safely be withheld in radiorecurrent prostate cancer when the results of combined PSMA PET and mpMRI are conclusive [33].

### 3.2. Use of Cryotherapy Post-Biochemical Recurrence after Radiotherapy

Salvage cryoablation may be a good alternative for patients with recurrent and residual localized disease. Radical prostatectomy for recurrent prostate cancer is associated with significant morbidity, including erectile dysfunction and high rates of stress incontinence, which makes the salvage cryoablation of radiation-resistant cancer a promising approach that may have fewer adverse outcomes [34]. Furthermore, these patients tend to be older and are therefore more likely to have medical comorbidities that increase perioperative risk of a major operation. In patients with biochemical recurrence, cryoablation can potentially offer curative treatment and functional preservation in the domains of urinary continence and erectile function, especially with salvage focal therapy [35,36].

When it comes to disease recurrence, large, bulky, high-grade, and bilateral tumors present a challenge to focal cryoablation. Leibovici et al. found that, among patients who had salvage radical prostatectomy after primary radiation therapy, 74% of patients experienced bilateral recurrence, one-third experienced multifocal recurrence, and 74% of tumors were located 5 mm from the urethra [37]. It is worth noting that most of the patients in the study were in the “pre-mpMRI” era and did not have post-radiation image-targeted biopsy before receiving salvage therapy. Nevertheless, one needs to take into account the characteristics of post-radiation recurrent prostate cancers, including site, size, location, and multifocality as shown by mpMRI and post-radiation biopsy, and pathology. The potential role of salvage focal therapy for radio-recurrent prostate cancer may therefore be a compelling reason for routine post-BCR prostate biopsy to assess candidacy for this approach. In this situation, it is critical to choose the right patients for targeted therapy.

- Whole-gland salvage cryotherapy

Whole-gland salvage cryoablation following primary radiotherapy failure has been the subject of several noteworthy studies. Table 4 lists these studies in detail for direct comparisons. Finley and Belldegrun (2011) reviewed the use of salvage cryotherapy as a viable treatment option with curative intent for radio-recurrent prostate cancer and found that complication rates trended downward with advances in technique and technology. Additionally, their review found that biochemical relapse-free rates ranged from 34% to 68% [38]. 

In a 2013 study, Spiess et al. found that the greatest predictors of biochemical progression-free survival (BPFS) following salvage cryotherapy were a pre-salvage biopsy Gleason score of 7 or lower and a post-cryoablation nadir PSA of less than 2.5 ng/mL. Furthermore, the biochemical recurrence-free survival (BRFS) rate was 45.5% at the 5-year mark [41]. The Cryo On-Line Database (COLD) registry revealed additional risk factors for failure, such as a castrate-resistant prostate cancer, prostate tumor stage of cT3–4, biopsy with a Gleason score over 8, and pre-cryotherapy PSA value greater than 10 ng/m [45]. In an earlier study by Spiess et al., risk for failure criteria was defined as a pre-cryotherapy PSA level of at least 5 ng/mL. Oncologic effectiveness rates were classified according to patients whose pre-salvage PSA level was less than 5 ng/mL and those whose pre-salvage PSA level was greater than 5 ng/mL. Biochemical disease-free survival (BDFS) was 52.9% for PSA > 5 ng/mL and 78.3% for PSA < 5 ng/mL at the 5-year mark [46].

Oncologic and functional outcomes of salvage cryotherapy post primary ERBT or cryotherapy were recently studied in a 2023 propensity score-matched analysis using the COLD registry and the Duke Prostate Cancer database. A total of *n* = 419 patients who underwent primary ERBT and experienced local recurrence, defined by a negative metastatic workup with conventional imaging (CT, bone scan, and/or MRI) and positive post-treatment prostate biopsy, were included in the study. Following a 72-month median follow-up, biochemical progression was observed in 55 (13.1%) and 90 (21.5%) of the patients at 2 and 5 years after salvage whole-gland cryotherapy, respectively [39]. Overall, *n* = 67 (16%) developed urinary incontinence defined as leakage requiring any number of pads, 11 (2.6%) developed fistulae, and 59 (14.1%) retained erectile function with or without pharmacologic intervention [39]. 

Ghafar et al. (2001) performed salvage cryosurgery on *n* = 38 patients with biopsy-proven recurrence following radiation therapy [44]. None of the patients had evidence of metastatic disease based on conventional staging imaging (CT and bone scans). All initially underwent neoadjuvant androgen deprivation treatment (NADT). After a median follow-up time of 20.7 months, the BRFS rate was 86% at 1 year and 74% at 2 years. Post-treatment complications included scrotal edema in 10.5% patients, urinary tract infection in 2.6%, incontinence in 7.9%, hematuria in 7.9%, and rectal pain in 39.5%. Urinary retention, urethral sloughing, or rectourethral fistulae did not occur in any patients (0%) [44].

In a study by Lian et al., salvage cryoablation was performed on *n* = 32 patients with biopsy-proven locally recurrent prostate cancer post-radiotherapy (*n* = 4 ERBT and *n* = 28 brachytherapy). All patients had negative metastatic screening with abdominal and pelvic MRI, as well as a whole-body bone scan. Patients with prostate glands > 60 mL and a prior history of a transurethral resection of the prostate were not offered cryotherapy. BRFS was defined as the time period from salvage treatment to the date of biochemical recurrence (Phoenix criteria of nadir +2 ng/mL), and complications were classified as grades 1–5 using the modified Clavien system. Mild incontinence was defined as requiring 1–2 pads per day after catheter removal. After a median follow-up of 63 months (range: 38–92), one patient experienced urethral sloughing, and three patients (3.1%) developed mild incontinence, which resolved within 6 weeks [43]. Of 14 patients (43.8%) who reported adequate erectile function before salvage treatment, 6 remained potent and 8 developed erectile dysfunction. The rate of a rectourethral fistula and urinary retention was 0%. Five-year overall survival was 92.3%, 5-year cancer-specific survival was 100%, and the 5-year BRFS rate was 43.5% [43].

A study conducted in 2013 by Wenske et al. included *n* = 396 patients who had prostate biopsies in addition to endorectal or pelvic 3T MRI to confirm recurrence. At the 5- and 10-year mark, they discovered that the BRFS rate following radiation whole-gland cryoablation was 63% and 37%, respectively [42]. Of the 328 patients who took part in the study, 11 had a second failure following radiation therapy (RT) and salvage cryotherapy (SC), and 20 patients (49%) experienced recurrence at the 20-month mark. In addition to other evidence of recurrence (such as radiography), failure was determined using the Phoenix criteria [42]. The 5- and 10-year disease-free survival rates were 47% and 42%, respectively, while the disease-specific survival rate was 100% and 83%, and overall survival was 87% and 81% [42].

Tan et al.’s 2023 study, which included *n* = 110 patients treated with salvage whole-gland cryoablation for biopsy-proven recurrence between 2002 and 2019, found that the BRFS at 12, 24, 36, 48, 60, and 72 months, respectively, was 81%, 79%, 75%, 71%, and 67% [40]. Pre-cryoablation PSA of 4–10 ng/dL (HR: 2.10, 95% CI: 1.00–4.41) and PSA of >10 ng/dL (HR: 4.26, 95% CI: 1.35–13.40) were linked to reduced BRFS, according to a multivariable Cox hazards analysis. Poorer BRFS was linked to a greater PSA nadir following salvage whole-gland cryoablation; in all patients, PSA nadir > 0.5 ng/mL ultimately resulted in biochemical recurrence [40]. 

Technology advancements in recent years have reduced the rate of complications related to salvage cryosurgery after EBRT. Notwithstanding these advancements, reports indicate that individuals undergoing salvage treatment had worse rates of pain and incontinence than those undergoing primary cryosurgery [5,47]. The retraumatization of previously damaged tissue has been documented as a complication as well [5]. Individuals who had an initial PSA of less than 10 ng/mL and a clinical stage T1-2N0M0 disease are more likely to benefit from salvage whole-gland cryotherapy for locally recurrent prostate cancer following EBRT. Following salvage therapy, these patients have greater percentages of negative biopsies, highlighting the efficacy of cryoablation for reliable and indelible tissue destruction [5]. In addition, two freeze–thaw cycles and a minimum of five cryoprobes should be considered to maximize the potential success of salvage whole-gland cryotherapy [48].

The outcomes of salvage whole-gland cryoablation provide a benchmark to which we can compare outcomes from salvage focal cryoablation studies.

b. Focal salvage cryotherapy

A number of studies have supported focal cryotherapy following radiation as an option with a relatively low number of adverse outcomes. These studies are delineated in Table 5.

Ismail et al. (2007) conducted a study of *n* = 100 patients who underwent targeted cryoablation. Although the planned treatment margin was not mentioned, the procedure was monitored by TRUS and four thermocouples were placed in the anterior prostate, the apex, Denonvilliers’ fascia, and the external sphincter. The study results found that 86% of patients developed erectile dysfunction, with other complications such as lower-urinary-tract symptoms (16%), incontinence (13%), prolonged perineal pain (4%), urinary retention (2%), and a rectourethral fistula (1%) [49]. Apart from these results, patients were also risk-stratified into low-, intermediate-, and high-risk groups according to their PSA level, Gleason score, and clinical stage before radiotherapy. The low-risk group consisted of patients with a PSA of ≤10 ng/mL, Gleason score of ≤6, and clinical stage ≤ T2b; the intermediate-risk group had one unfavorable factor from a PSA of >10 ng/mL, Gleason score of ≥7, and clinical stage > T2b; and the high-risk group had two or more unfavorable risk factors [49]. At a 5-year follow-up mark, it was found that BRFS survival for groups was 11% for high-risk, 45% for intermediate-risk, and 73% for low-risk groups [49]. This study defined BRFS as cases that met the ASTRO criteria and had a PSA of <0.5 ng/mL after salvage targeted cryoablation. It should also be noted that for this categorization, all patients were re-staged following cryoablation with bone scans, pelvic MRI, and prostate biopsy as indicated. The study supported targeted cryoablation as a viable alternative for recurrent prostate cancer but noted that the high reported rate of erectile dysfunction was an adverse outcome to consider in the context of patient preferences.

**Table 5:** Functional and oncological outcome parameters for salvage focal cryoablation studies.

References	Institution	Initial Therapy (No. of Pts)	Risk Categories	Preprocedural PSA (ng/mL), Median (IQR); Use of ADT, *n* (%)	Mos. Follow-Up	Focal Template; Planned Treatment Margin	Success/ Failure Criteria ^a^	% Post-Ablative Biopsy; % Pos. Biopsy Rate	Urinary Incontinence, *n* (%); Erectile Function, *n* (%)
Li et al. (2015) [50]	Cleveland Clinic, OH, USA	RT (91)	NR	4.8 (0–92.6); 0 (0%)	Median: 15	NR; NR	BDFS at 1 year: 95.3%, 3 years: 72.4%, 5 years: 46.5%	15.4%; 28.6%	5 (5.5); 10 (50)
Kongnyuy et al. (2017) [51]	Winthrop University Hospital, Garden City, NY, USA	CR—8 (12.3%) SR—5 (7.7) BT—13 (20.0) PBR—1 (1.5) RT/other—37 (57.0) Unknown—1 (1.5) (*n* = 65)	NR	4.0 (0.01–19.0); 13 (20%)	Median: 26.6	Hemi; NR	BR: 52.3%	52.3%; 20%	4 (6.1); 14 (21.5)
Tan et al. (2021) [52]	Duke University Medical Center, Durham, NC, USA	NR (11)	NR	4.99 (2.23–7.86); 0 (0%)	Median: 28	Focal (*n* = 6); NR, Hemi (*n* = 2); NR, Sub-total (*n* = 3); NR	FFS at 12 months: 100%, 24 months: 80%, and 36 months: 40%	NR; 27.3%	1 (0.1); NR
Tan et al. (2020) [53]	Multiple, USA	RT (72)	NR	4 (2.7–5.6); 19 (26.4%)	Median: 24.4	NR; NR	BR: 16 of the 72 patients (22.2%)	19.2%; 33.3%	9.3%; 52.6%
Ismail et al. (2007) [49]	The Royal Surrey County Hospital and St Luke’s Cancer Centre, Guildford, Surrey, UK	RT (100)	High: 68, Intermediate: 20, Low: 12	NR; NR	33.5	NR; NR	BRFS at 5 years: 73% (low-risk), 45% (intermediate), and 11% (high)	NR; NR	13, ED: 86
Chang et al. (2015) [54]	The Affiliated Hospital of Nanjing University Medical School, Jiangsu, China	CR (12)	NR	2.5 (0.18–7.28); 3 (25%)	Median: 33.5		BRFS: 7 (58.3%)	16.7; 16.7	1 (8.3), impotence: 2 (16.6)
Bomers et al. (2013) [55]	Multiple (the Netherlands)	CR (10)	NR	3.6 (0.9–8.7); NR	12	NR; NR	BRFS at 3 months: 100%; BRFS at 6 months: 70%; BRFS at 12 months: 75% [3/4]	NR; NR	NR; NR
de Abreu Castro (2013) [56]	Multiple (USA)	SFC (25), STC (25)	NR	2.8 (SFC); 3.9 (STC)	31 months (SFC); 53 months (STC)	NR; NR	BFS at 5 years: 54% (SFC), 86% (STC)	48% (SFC), 28% (STC); 14.3% [1/7] (STC)	0% (SFC), 13% (STC); 29% [2/7] (SFC), 0% [0/4] (STC)

^a^ Biochemical recurrence defined using the Phoenix criteria; exception: Ismail et al. (ASTRO definition). RT  =  radiotherapy; NR = not reported; BRFS = biochemical recurrence-free survival; BDFS = biochemical disease-free survival; FFS = failure-free survival; BR = biochemical recurrence; ED = erectile dysfunction; CR = cryotherapy; SR = stereotactic radiotherapy; BT = brachytherapy; PBR = proton beam radiation; SFC = salvage focal cryoablation; STC = salvage total cryoablation; Hemi = hemiablation.

In another study by Li et al. (2015), researchers utilized the Cryotherapy On-Line Data (COLD) registry to examine outcomes of salvage cryotherapy for local recurrent prostate cancer. A total of *n* = 91 patients were identified and had biochemical disease-free survival rates at 1, 3, and 5 years of 95.3%, 72.4%, and 46.5%, respectively [50]. Failure was defined using the Phoenix criteria (PSA nadir + 2 ng/mL). Of those patients who underwent biopsy after the salvage treatment, 28.6% [4/14 patients] had local failure. Researchers also measured incontinence, defined as using pads, and potency, defined as the ability to have intercourse. It was found that 5 patients (5.5%) reported incontinence over a 12-month period, and 10 patients (50%) remained potent after salvage treatment. Other complications of this therapy included urinary retention in six patients (6.6%), transurethral resection in one patient (1.1%), and a rectourethral fistula in three patients (3.3%) [50].

A fourth study by Bomers et al. (2013) examined the feasibility of multiparametric, magnetic resonance-guided focal cryoablation in *n* = 10 patients who had undergone radiation therapy. After a 6-month follow-up, *n* = 2 patients were found to have a local recurrence [55]. By the 12-month mark, *n* = 3 patients in total were found to have recurrences. These recurrences were defined as both an increased PSA level and clinician suspicion on MR images, which the authors attributed to an overly conservative estimation for the area of focal cryoablation. Patient-reported outcomes included *n* = 1 urethral stricture, *n* = 2 urinary retention, and *n* = 1 each of hematospermia, hematuria, and perineal pain cases [55]. The study’s authors concluded that the MR imaging-guided focal cryoablation proved to be a reasonable option for recurrent prostate cancer, noting the relatively limited sample size and shorter follow-up time.

In a more recent study by Kongyung (2017), *n* = 65 patients also underwent salvage focal cryotherapy. However, the study examined focal treatment in the context of whether or not it could delay use of ADT. At the time of study publication, 80% of patients (*n* = 52) had not received ADT [51]. Upon the selection of patients, it was reported that 86.2% had radiation therapy as primary treatment. Upon the follow-up of biochemical-free survival, 48.1% of patients had survived at the 1-year and 3-year mark. Patient-reported outcomes included urethral strictures (4.1%, *n* = 3), incontinence (21.5%, *n* = 14), and erectile dysfunction (21.5%, *n* = 14) [51]. Authors concluded that salvage focal cryotherapy was promising in delaying the use of ADT.

In a 2021 retrospective study, Tan et al. evaluated the oncological and functional outcomes of *n* = 11 patients who underwent salvage partial-gland cryoablation. Failure-free survival was defined as the absence of infield or out-of-field recurrence, absence of whole-gland or systemic therapy, and absence of mortality. Metastasis-free survival was defined as the duration from the date of treatment to the last follow-up or the diagnosis of metastasis on a bone scan or PET/CT as indicated by the clinician. At 12, 24, and 36 months, failure-free survival was 100%, 80%, and 40%, respectively, whereas metastasis-free survival was 100%, 75%, and 50%, at the same intervals [52]. Success of partial-gland cryoablation was defined by three factors: (1) the ability to have penetrative sex post-treatment; (2) remaining continent and pad-free; and (3) not having any infield or out-of-field recurrence, and being able to avoid whole-gland or systemic treatment and death related to prostate cancer post-treatment. Results showed that 79% of patients were able to achieve all three factors [52].

### 3.3. Post-Focal Ablation Recurrence

Multiple studies report decreases in localized prostate cancer recurrence rates with focal ablation treatment. Studies report up to 60–94% disease-free rates at post-ablation biopsy. Notably, the identified areas of recurrence were often in untreated areas of the prostate [57].

In a 2023 study, Campbell et al. examined the use of salvage cryotherapy for local prostate cancer recurrence following primary radiotherapy or primary cryotherapy. The results showed no difference in BPFS or in functional outcomes between the two cohorts (post-radiotherapy or post-prior cryotherapy) [39]. Additionally, BPFS was lower in D’Amico high-risk and intermediate-risk groups than in low-risk groups. These results indicate that the use of salvage focal cryoablation is as beneficial in treating primary cryotherapy recurrence as it is for primary radiotherapy recurrence [39]. The majority of studies on focal cryoablation in the salvage setting examine its use for radiotherapy recurrence treatment; this study adds to the literature on salvage focal cryotherapy by showing its effectiveness against primary cryotherapy recurrence as well. 

In a 2019 retrospective study, Aminsharifi et al. examined the effects of salvage cryotherapy in *n* = 108 patients who had biopsy-proven local recurrence of prostate cancer following previous cryotherapy. In order to maximize oncological control and minimize therapeutic harm, patients underwent either whole-gland or focal cryotherapy, based on the distribution of positive cores seen on biopsy. Some patients received ADT (32.4%), and some received radiotherapy (21.3%), and others received salvage cryotherapy alone. The oncological outcome was defined by the rate of biochemical recurrence after salvage ablation using Phoenix criteria. After two and five years, biochemical rates were 28.2% and 48.3%, respectively [58]. Use of ADT or radiotherapy before salvage treatment and use of focal versus whole-gland treatment were not significant predictors of biochemical recurrence. In terms of functional outcomes, urinary incontinence was reported in 7.4% of patients one year following salvage treatment, with higher persistent incontinence rates for patients who received radiation before salvage treatment as compared to those who did not (21.7% vs. 3.5%) [58]. Only 13.8% of patients were able to have spontaneous or medically assisted erections suitable for intercourse following salvage treatment. Temporary urinary retention was reported in 3.7% of patients. A rectourethral fistula was also reported in 3.7% of patients, all of whom, notably, had high-risk disease and received whole-gland treatment [58].

In a 2015 retrospective study, Chang et al. similarly examined the effects of salvage cryotherapy in *n* = 12 patients with locally recurrent prostate cancer following primary cryotherapy. Patients were seen every 3 months for a physical examination, PSA measurement, and radiologic imaging when clinically indicated. The median PSA level prior to salvage treatment was 2.5 ng/mL, as compared to 1.32 ng/mL PSA nadir following salvage treatment [54]. Following salvage treatment, two patients received hormonal therapy and two received repeat cryotherapy. Functional outcomes following salvage treatment included mild incontinence in one patient, urethral sloughing in one patient, and transient impotence in two patients. The use of salvage cryotherapy allowed for the delay of hormonal therapy [54]. The results of this study indicate the safe and effective use of salvage cryotherapy for recurrent prostate cancer following primary cryotherapy. 

## 4. Comparison of Salvage: Partial vs. Salvage Whole-Gland Cryotherapy

Salvage cryotherapy results in less morbidity than whole-gland treatment and offers oncological outcomes comparable to salvage radical prostatectomy and whole-gland ablative treatment [52,59]. In carefully chosen patients, a targeted salvage approach may lower the risk of adverse effects while maintaining cancer control. There are few studies comparing focal cryotherapy to whole-gland cryotherapy within a single cohort. 

Tan et al. (2020) conducted a retrospective review using the COLD registry and found that, when compared to patients treated with salvage total cryoablation (STC; *n* = 313) for biopsy-proven radiation therapy-resistant disease, *n* = 72 patients treated with salvage focal cryotherapy (SFC) had a lower risk of urinary retention [53]. A rectourethral fistula was only observed in the whole-gland salvage group (*n* = 4); and spontaneous or medication-augmented erections sufficient for intercourse were observed in *n* = 15 patients, irrespective of treatment group [53]. There were no statistically significant differences in 2-year progression-free survival and post-treatment biopsy cancer control rates. The lack of imaging data in the COLD registry prevented the study’s authors from identifying the variables that affected clinicians’ choices between SFC and STC treatment. These findings imply that although side effects are lower with targeted cryotherapy, oncologic outcomes for salvage whole-gland therapy are comparable.

de Castro Abreu et al. (2013) compared outcomes of *n* = 50 patients who underwent salvage focal cryotherapy (*n* = 25) and total cryoablation (*n* = 25) after failed primary radiotherapy. Salvage focal cryotherapy was defined in the study as a hemiablation of the targeted prostate lobe. Biochemical failure was defined using the Phoenix criteria of the PSA nadir +/− 2 mg/mL and occurred in 32% of patients with salvage focal cryotherapy and 12% of salvage total cryotherapy patients [56]. The study reported a 5-year biochemical-free survival rate of 54.4% of salvage focal cryotherapy patients. In terms of patient-reported outcomes, there were no patients in the salvage focal group that developed incontinence, compared to 13% in the salvage total cryotherapy group. Twenty-nine percent of patients in the salvage focal group maintained potency, compared to none of the patients in the salvage total group. There was also one patient in the salvage total group that reported development of a rectourethral fistula [56].

In the study by Wenske et al. that was discussed in the section on whole-gland salvage cryotherapy, *n* = 273 underwent total cryotherapy (STC), and *n* = 55 patients had focal cryotherapy (SFC). After a median follow-up of 47.8 months, 42 patients in the STC group (15.4%) and 4 in the SFC group (7.3%) experienced treatment complications [42]. Within the STC group, there were 15 (5.5%) urethral strictures, and 10 (3.7%) bladder outlet obstructions requiring TURP or the photoselective vaporization of the prostate (PVP). There were seven (2.6%) patients with acute urinary retention, seven (2.6%) with incontinence, and six (1.8%) with rectourethral/rectovesical fistulae. There were only three (5.5%) with a rectourethral/rectovesical fistula and one (1.8%) with a bladder outlet obstruction requiring TURP or PVP in the SFC group [42]. It was concluded that patients with preserved erectile function and full continence before salvage cryotherapy may benefit the most from focal treatment.

## 5. Comparison of Salvage Cryotherapy vs. Salvage Radical Prostatectomy

Patients with recurrent disease following radiotherapy may be candidates for salvage prostatectomy; however, this procedure is more challenging than primary surgery or cryotherapy due to periprostatic fibrosis, and thus associated with higher complication rates such as incontinence, erectile dysfunction, rectal injury, and positive surgical margins [60]. Furthermore, cavernous nerve sparing is more difficult during a salvage prostatectomy. Keeping these in mind, this section will evaluate the few studies that compared the oncologic and/or functional outcomes of salvage radical prostatectomy versus salvage cryotherapy.

In a 2009 retrospective study, Pisters et al. compared patients who underwent salvage radical prostatectomy (SRP) at the Mayo Clinic between 1990 and 1999, and those who underwent salvage cryotherapy (SCT) at M.D. Anderson Cancer Center between 1992 and 1995. In the SRP group, the prostate gland, periprostatic tissues (including neurovascular bundles), and the semilunar vesicles were completely removed. There was no mention of the positive surgical margin rate. All patients had a PSA < 10 ng/mL and post-radiation therapy biopsy-proven prostate cancer with a Gleason score < 8; none of the patients received pre- or post-salvage hormonal therapy [61]. The criteria for biochemical disease-free survival (BDFS) were a PSA > 0.4 ng/mL and two increases above the nadir PSA. After a mean follow-up of 7.8 years for the SRP group and 5.5 years for the SCT group, SRP resulted in superior BDFS by both definitions of biochemical failure. BDFS for PSA > 0.4 ng/mL was 21% in the SCT group versus 61% in the SRP group at 5 years (*p* < 0.001). DBFS for two increases above nadir was 42% in the SCT group versus 66% in the SRP group at 5 years (*p* = 0.002) [61]. Five-year disease-specific survival did not differ significantly between the two groups (96% for SCT vs. 98% for SRP, *p* = 0.283). The tumor grade was significantly higher in the SCT group, potentially confounding the results given the association between a high tumor grade and PSA failure following SCT [61]. Functional outcomes were not reported in the study. 

Vora et al. (2016) compared the outcomes of salvage cryotherapy versus salvage robotic prostatectomy for patients with radio-resistant disease at Cleveland Clinic Florida between 2004 and 2013. A total of 23 salvage procedures were performed: *n* = 6 patients underwent salvage prostatectomy, and *n* = 17 underwent salvage cryotherapy. Patients with localized disease at presentation, a PSA < 10 ng/mL at recurrence, and a life expectancy > 10 years at recurrence were considered for salvage treatment [62]. Both salvage options were presented, and patient preference was used as the deciding factor. The cutoff value for biochemical recurrence following primary salvage therapy was two subsequent rises in PSA more than 6 months after reaching nadir. After a modest mean follow-up of 14.1 months and 7.2 months, the incidence of disease progression was 23.5% and 16.7% after salvage cryotherapy and prostatectomy, respectively [62]. The overall complication rate was 23.5% after salvage cryotherapy versus 16.7% after salvage prostatectomy. Furthermore, the most common complication following salvage cryotherapy was urethral stricture (11.8%), whereas severe urinary incontinence (16.7%) was the most common complication following salvage prostatectomy. In the salvage cryotherapy group, one patient developed a rectourethral fistula [62].

Using the Surveillance, Epidemiology, and End Results (SEER) database, Friedlander et al. (2014) found that salvage radical prostatectomy was associated with higher overall mortality in Medicare beneficiaries over 65 [63]. Of a total of 440 men who were retrospectively identified between 1992 and 2009, *n* = 341 underwent salvage cryotherapy (SCT), and *n* = 99 underwent salvage radical prostatectomy (SRP). Median follow-up post-salvage therapy was 30 and 15 months for SRP and SCT, respectively. Overall mortality was higher for the SRP group versus the SCT group (21.6 vs. 6.1 deaths/100 person years, *p* < 0.001) [63]. There was also a trend toward higher prostate cancer-specific death rates for the SRP group versus the SCT group (6.5 vs. 1.4 deaths/100 person years, *p* = 0.061) [63].

The tumor grade was not significantly higher in one group versus the other (*p* = 0.134). Notably, compared to the SRP group, those who received SCT were more likely to be white (*p* < 0.001), reside in areas with <85% high school graduates (*p* = 0.008), and have previously received ADT (50.4% vs. 45.5%, *p* = 0.001) and primary brachytherapy (43.7% vs. 24.2%, *p* = 0.001) [63]. Furthermore, the median primary to salvage therapy time interval was significantly shorter in the SCT group compared to the SRP group (38.7 months vs. 55.8 months, *p* < 0.001). The results of this study are susceptible to the confounding effect of hormonal therapy because the temporal variability of salvage ADT was not controlled for [63]. Additionally, potential differences in disease severity related to longer primary to salvage therapy durations may not have been taken into account in the study’s adjusted analysis. The authors also acknowledge that there might be selection bias in favor of young men to undergo SRP because of less comorbidities and postoperative incontinence compared to older men. 

## 6. Comparison of Salvage Cryotherapy vs. Salvage High-Intensity Focused Ultrasound

Salvage high-intensity focused ultrasound (HIFU) and salvage cryotherapy have emerged in treating recurrent, localized prostate cancer. In a study by Autran-Gomez et al., it was found that both HIFU and cryotherapy offered promising salvage treatment options but that there is a significant risk of complications as well as lack of general consensus on treatment recommendations [64].

One of the factors contributing to the lack of recommendations on usage of these modalities is the absence of a consensus for what constitutes proper PSA response/nadir after salvage therapy (RT). Due to the nature of salvage HIFU and cryoablation, which are both ablative procedures, it is important to take into account other factors such as benign PSA secreting tissue, which may impact perceived efficacy. As a result, many rely on the use of prostate biopsy at 6–12 months following salvage treatment to indicate the possibility of a recurrence [64]. In addition, this time frame allows for PSA nadir to be reached, if at all. In a study by Uchida et al., it was shown that patients who underwent salvage HIFU and had a PSA nadir of below 0.2 ng/mL had a low rate of cancer detection compared to those whose PSA nadir was above 0.21 [65]. Furthermore, imaging such as contrast-enhanced MRI may serve as another postoperative monitoring option in the detection of local recurrence after salvage HIFU with a sensitivity and specificity of 0.98 and 0.81, respectively [66].

Unlike the relatively sparse literature pertaining to salvage HIFU therapy, salvage cryotherapy is seen as a more established option in the salvage setting, with FDA approval for primary cryoablation attained around 1999 [64]. As mentioned above, there is no standard defined failure criteria for measuring salvage cryotherapy; however, salvage cryotherapy offers the option for repeat ablation with the caveat and caution of increased risk of fistula formation and incontinence [64]. In these patient populations who may be at risk for recurrence, PSA appears to be a stronger predictor of recurrence. In a separate study that analyzed disease-free survival after salvage cryotherapy treatment methods, patients with a pre-salvage PSA of >10 ng/mL were considered poor candidates and had higher rates of treatment failure if they were unable to attain a PSA nadir of <1 ng/mL [67]. It should also be noted that because this particular study examined patients who underwent salvage cryotherapy between 1995 and 2004, it was not mentioned whether or not patients benefited from PSMA PET staging as the technology used in this context was newly developing [68].

In the salvage setting, cryotherapy may offer an advantage over HIFU, particularly in cases of prior prostate brachytherapy or significant intraprostatic calcifications. Post-radiation intraprostatic calcifications can create acoustic shadowing and a reflection of sound waves that create dangerous “pre-focal heat” during salvage HIFU, increasing the risk of devastating rectourethral fistulae. Currently, there is sparse literature comparing the efficacy of salvage cryotherapy to salvage HIFU for the treatment of radio-recurrent prostate cancer, both in the whole-gland and focal setting. Nevertheless, specific treatment recommendations are emerging as alternatives for patients who have contraindications to salvage radical prostatectomy, which results in high functional morbidity. Patients who may be suitable candidates for salvage cryotherapy or HIFU must be informed about the increased risk of complications as well [64]. 

The functional and oncological outcomes for salvage whole-gland and focal cryoablation studies are summarized in Table 4 and Table 5, respectively.

## 7. Limitations

There were some limitations within this narrative review, namely the small size of some studies and limited follow-up time in some of the cited research. Significant efforts were made to include a comprehensive review of the literature on this topic, including an extensive literature review.

## 8. Conclusions 

Salvage cryotherapy is a relatively safe and effective treatment option for recurrent prostate cancer and may even allow for hormonal therapy to be delayed. Focal cryotherapy, in particular, results in lower morbidity than whole-gland treatment and has yielded oncological outcomes comparable to salvage radical prostatectomy and whole-gland ablative therapy in several small retrospective series. Additionally, results from a recent study suggest that oncologic outcomes for salvage whole-gland and focal cryotherapy are similar, emphasizing the potential for an improved morbidity profile with focal therapy.

Despite improvements, the retraumatization of previously damaged tissue was found to be associated with complications. Pain and incontinence rates in the salvage setting, for example, were reported to be higher than in patients who underwent primary cryosurgery. Nonetheless, results from the contemporary literature pointed to a low incidence of complications and a high percentage of favorable oncologic outcomes, indicating that salvage cryotherapy may be useful in treating recurrent prostate cancer for properly selected patients, such as those with a pre-salvage treatment PSA < 5 ng/mL or 10 ng/mL, Gleason score of 7 or less at recurrence, and absence of locally advanced (i.e., T3–4) disease at recurrence. Further meta-analyses, and prospective updates from newly accrued data from the ongoing COLD registry, are certainly necessary to help to inform selection criteria for whole-gland vs. focal therapy and to validate the conclusions of the published series to date. 

Clinical trials are seeking to evaluate the impact of gene expression data in prostate cancer care, and determine how genomic information from individual patients can be used in population-level analyses to improve treatment and outcomes. Integrating these gene expression classifiers (i.e., Decipher biopsy) could refine therapeutic decisions following salvage cryotherapy, aligning with emerging personalized treatment paradigms and potentially enhancing long-term outcomes [69]. The role of gene expression classifiers, as well as treatment intensification with systemic therapy such as androgen deprivation therapy, remains the subject of further study. Other suggestions for future research could include trials focused on salvage cryotherapy with PSMA PET scans or genomic testing and/or gene expression classifiers. Additionally, previous studies have reported that focal cryotherapy and stereotactic body radiotherapy (SBRT) are associated with similar oncologic and functional outcomes [70,71]. These outcomes should be further investigated for radio-recurrent prostate cancer in the salvage setting.

The patient’s priorities and expectations, especially with regard to functional outcomes of urinary continence, erectile function, and other measures of quality of life, should be taken into account when selecting cryotherapy as a salvage treatment option. 

## Figures and Tables

**Table 1:** Diagnostic performance PSMA scans for predicting biochemical recurrent prostate cancer.

References	Institution(s)	Prior Treatment (No. of Patients)	Preprocedural PSA (ng/mL), Median (IQR)	Site of Failure	Predictive Factors	PPV
Hruby et al. (2017) [17]	Royal North Shore Hospital, Australia	EBRT (*n* = 419)	Median (range): 10 (1–145)	Local only: 8 (16.7%) Local and distant: 15 (31.3%) Distant only: 25 (52%)	Gleason scores ≥ 8 (17% vs. 9%, *p* = 0.011) and initial PSA > 10 ng/mL (16% vs. 8%, *p* = 0.04)	NR
Einspieler et al. (2017) [18]	Multiple (Germany, USA)	EBRT: 77 BT: 41 (*n* = 118)	>Median: 10.7 (6.9–24.7)	Local only: 43 (40.2%) Local and distant: 25 (23.4%) Distant only: 39 (36.4%)	Increasing PSA and concomitant ADT (SUV_max_: *p* = 0.018 and 0.004; SUV_mean_: *p* = 0.025 and 0.007, respectively)	NR
Morris et al. (2021) [19]	Multiple (USA)	*n* = 208	Median (range): 0.8 (0.2–98.4)	NR	NR	Prostatic region: 75.0–83.3% (range) Pelvic lymph nodes: 67.2–72.7% Extra-pelvic region: 67.3–69.8%

PPV = positive predictive value; NR = not reported; BT = brachytherapy.

**Table 2:** Diagnostic performance of different combinations of MRI for predicting locally recurrent prostate cancer.

MRI	Sensitivity (%)	Specificity (%)	PPV (%)	NPV (%)	AUC (%)
Kim et al. (2009) [25], Sungkyunkwan University School of Medicine, Republic of Korea					
T2W	25	92	57	74	61.2
T2W + DWI	62	97	91	81	87.9
Kim et al. (2010) [26], Kangwon National University College of Medicine, Republic of Korea					
DWI	49	93	72	84	78.2
DCE	49	92	67	84	73.7
DWI + DCE	59	91	69	87	86.3
T2W	27	80	32	76	59.4
Tamada et al. (2011) [27], Kawasaki Medical School, Japan					
mpMRI	77	92	68	95	NR
T2W	27	99	86	87	NR
DCE	50	98	85	90	NR
DWI	68	95	75	94	NR

PPV = positive predictive value; NPV = negative predictive value; AUC = area under the curve; NR = not reported.

**Table 3:** Diagnostic performance of PSMA PET vs. mpMRI for predicting recurrent prostate cancer.

Imaging	Sensitivity (%)	Specificity (%)	PPV (%)	NPV (%)	Accuracy (%)
Radzina et al. (2020) [28], Multiple (Latvia, Germany)					
PSMA PET (local)	63.6	73.7	58.3	77.8	77.8
mpMRI (local)	90.9	94.7	90.9	94.7	92.3
PSMA PET (LN)	83.3	80.0	80	100	90.6
mpMRI (LN)	41.7	94.4	83.3	70.8	72.0
PSMA PET (bone)	83.3	92.0	71.4	95.8	71.0
mpMRI (bone)	50.0	84.0	42.8	87.5	77.4
Jannusch et al. (2023) [31], Multiple, Germany					
PSMA PET (local)	50	96	50	96	93
mpMRI (local)	100	100	100	100	100
PSMA PET (LN)	96	97	97	97	97
mpMRI (LN)	57	92	81	78	79
PSMA PET (dLN)	50	100	100	98	98
mpMRI (dLN)	0	100	-	97	97
PSMA PET (bone)	100	100	100	100	100
mpMRI (bone)	67	98	80	96	95

PPV = positive predictive value; NPV = negative predictive value; LN = lymph node metastases; dLN = distant lymph node metastases.

**Table 4:** Functional and oncological outcomes for salvage whole-gland cryoablation studies.

References	Institution	Initial Therapy (No. of Pts)	Risk Categories	Preprocedural PSA (ng/mL), Median (IQR)	Mos. Follow-Up	Use of ADT, *n* (%)	Success/Failure Criteria ^a^	% Pos. Biopsy Rate	Urinary Incontinence, *n* (%); Erectile Function, *n* (%)
Campbell et al. (2023) [39]	Duke University Medical Center, Durham, NC, USA	ERBT (419)	D’Amico Low: 120 (28.6%), Intermediate: 140 (33.4%), High: 159 (37.9%)	Mean: 7.01	Median: 72 (60–170)	142 (33.9%)	Biochemical progression at 2 years: 55 (13.1%), and 5 years: 90 (21.5%)	NR	67 (16%); 59 (14.1%)
Tan et al. (2023) [40]	Duke University Medical Center, Durham, NC, USA	RT (110)	NR	Median: 3.87 (2.48–5.86)	Median: 71 (42.3–116)	22 (20%)	BRFS at 12 months: 85%, 24 months: 81%, 36 months: 79%, 48 months: 75%, 60 months: 71%, and 72 months: 67%	NR	9%; NR
Spiess et al. (2013) [41]	Department of Genitourinary Oncology, Tampa, FL	NR (132)	D’Amico Low: 18 (14%), Intermediate: 82 (62%), High: 32 (24%)	Mean: 6.2 (4.9–34.2)	Mean (range): 4.0 (0.9–12.7)	0 (0%)	bPFS at 1 year: 87.8%, 2 years: 72.4%, and 5 years: 45.5%	NR	NR
Wenske et al. (2013) [42]	Multiple, USA	EBRT (259), BT (49), CR (20)	NR	Median (range): 8 (0.6–290)	Median (range): 47 (1.6–203.5)	NR	BRFS at 5 years: 63%, 10 years: 37%	NR	7 (2.1); NR
Lian et al. (2016) [43]	Department of Urology, Nanjing University, China	ERBT (4), BT (28)	NR	Median: 7.9 (3.2–17.6)	Median: 63 (38–92)	5 (15.6%)	BRFS at 5 years: 43.5%	NR	
Ghafar et al. (2001) [44]	Department of Urology, Columbia University, New York, USA	EBRT (38)	NR	Mean: 7.5	Mean (range): 20.7 (3–37)	38 (100%)	BRFS at 1 year: 86%, at 2 years: 74%	NR	3 (7.9); NR

^a^ Biochemical recurrence defined using the Phoenix criteria; exception: Ghafar et al. (>0.3 ng/mL above the PSA nadir). RT  =  radiotherapy; CR = cryotherapy; EBRT = external beam radiation therapy; BT = brachytherapy; NR = not reported; BRFS = biochemical recurrence-free survival; bPFS = biochemical progression-free survival.

## References

[1] American Cancer Society (2024). Cancer Facts & Figures 2024.

[2] Kishan A.U., Karnes R.J., Romero T., Wong J.K., Motterle G., Tosoian J.J., Trock B.J., Klein E.A., Stish B.J., Dess R.T. (2021). Comparison of Multimodal Therapies and Outcomes Among Patients with High-Risk Prostate Cancer with Adverse Clinicopathologic Features. JAMA Netw. Open.

[3] Chin J.L., Lavi A., Metcalfe M.J., Siddiqui K., Dewar M., Petros F.G., Li R., Nogueras González G.M., Wang X., Nair S.M. (2021). Long-Term Outcomes of Whole Gland Salvage Cryotherapy for Locally Recurrent Prostate Cancer following Radiation Therapy: A Combined Analysis of Two Centers. J. Urol..

[4] Zaorsky N.G., Calais J., Fanti S., Tilki D., Dorff T., Spratt D.E., Kishan A.U. (2021). Salvage therapy for prostate cancer after radical prostatectomy. Nat. Rev. Urol..

[5] Safavy S., Jabaji R.B., Lu S.M., Slezak J.M., Cosmatos H.A., Williams S.G., Finley D.S. (2019). Salvage Cryoablation for Radiorecurrent Prostate Cancer: Initial Experience at a Regional Health Care System. Perm. J..

[6] Khan A., Khan A.U., Siref L., Feloney M. (2023). Focal cryoablation of the prostate: Primary treatment in 163 patients with localized prostate cancer. Cureus.

[7] Tan W.P., Wysock J.S., Lepor H. (2023). Partial gland cryoablation for prostate cancer—Where are we?. Nat. Rev. Urol..

[8] Sonn G.A., Margolis D.J., Marks L.S. (2014). Target detection: Magnetic resonance imaging-ultrasound fusion-guided prostate biopsy. Urol. Oncol..

[9] Valerio M., Shah T.T., Shah P., Mccartan N., Emberton M., Arya M., Ahmed H.U. (2017). Magnetic resonance imaging-transrectal ultrasound fusion focal cryotherapy of the prostate: A prospective development study. Urol. Oncol Semin. Orig..

[10] Priester A., Natarajan S., Khoshnoodi P., Margolis D.J., Raman S.S., Reiter R.E., Huang J., Grundfest W., Marks L.S. (2017). Magnetic resonance imaging underestimation of prostate cancer geometry: Use of patient specific molds to correlate images with whole Mount Pathology. J. Urol..

[11] Littrup P.J., Jallad B., Vorugu V., Littrup G., Currier B., George M., Herring D. (2009). Lethal isotherms of cryoablation in a phantom study: Effects of heat load, probe size, and number. J. Vasc. Interv. Radiol..

[12] Shah T.T., Arbel U., Foss S., Zachman A., Rodney S., Ahmed H.U., Arya M. (2016). Modeling cryotherapy ice ball dimensions and isotherms in a novel gel-based model to determine optimal cryo-needle configurations and settings for potential use in clinical practice. Urology.

[13] de Marini P., Cazzato R.L., Garnon J., Shaygi B., Koch G., Auloge P., Tricard T., Lang H., Gangi A. (2019). Percutaneous MR-guided prostate cancer cryoablation technical updates and literature review. BJR Open.

[14] Selvaggio O., Falagario U.G., Bruno S.M., Recchia M., Sighinolfi M.C., Sanguedolce F., Milillo P., Macarini L., Rastinehad A.R., Sanchez-Salas R. (2021). Intraoperative digital analysis of ablation margins (DAAM) by fluorescent confocal microscopy to improve partial prostate gland cryoablation outcomes. Cancers.

[15] Chin Y.F., Lynn N. (2022). Systematic review of focal and salvage cryotherapy for prostate cancer. Curēus.

[16] Agarwal P.K., Sadetsky N., Konety B.R., Resnick M.I., Carroll P.R. (2008). Treatment failure after primary and salvage therapy for prostate cancer: Likelihood, patterns of care, and outcomes. Cancer.

[17] Hruby G., Eade T., Kneebone A., Emmett L., Guo L., Ho B., Hsiao E., Schembri G., Hunter J., Kwong C. (2017). Delineating biochemical failure with 68Ga-PSMA-PET following definitive external beam radiation treatment for prostate cancer. Radiother. Oncol..

[18] Einspieler I., Rauscher I., Düwel C., Krönke M., Rischpler C., Habl G., Dewes S., Ott A., Wester H.-J., Schwaiger M. (2017). Detection Efficacy of Hybrid 68Ga-PSMA Ligand PET/CT in Prostate Cancer Patients with Biochemical Recurrence after Primary Radiation Therapy Defined by Phoenix Criteria. J. Nucl. Med..

[19] Morris M.J., Rowe S.P., Gorin M.A., Saperstein L., Pouliot F., Josephson D., Wong J.Y., Pantel A.R., Cho S.Y., Gage K.L. (2021). Diagnostic Performance of 18F-DCFPyL-PET/CT in Men with Biochemically Recurrent Prostate Cancer: Results from the CONDOR Phase III, Multicenter Study. Clin. Cancer Res..

[20] Perera M., Papa N., Roberts M., Williams M., Udovicich C., Vela I., Christidis D., Bolton D., Hofman M.S., Lawrentschuk N. (2020). Gallium-68 Prostate-specific Membrane Antigen Positron Emission Tomography in Advanced Prostate Cancer-Updated Diagnostic Utility, Sensitivity, Specificity, and Distribution of Prostate-specific Membrane Antigen-avid Lesions: A Systematic Review and Meta-analysis. Eur. Urol..

[21] Mertan F.V., Greer M.D., Borofsky S., Kabakus I.M., Merino M.J., Wood B.J., Pinto P.A., Choyke P.L., Turkbey B. (2016). Multiparametric Magnetic Resonance Imaging of Recurrent Prostate Cancer. Top. Magn. Reson. Imaging.

[22] Gaur S., Turkbey B. (2018). Prostate MR Imaging for Posttreatment Evaluation and Recurrence. Radiol. Clin. N. Am..

[23] Barchetti F., Panebianco V. (2014). Multiparametric MRI for recurrent prostate cancer post radical prostatectomy and postradiation therapy. Biomed. Res. Int..

[24] Grant K., Lindenberg M.L., Shebel H., Pang Y., Agarwal H.K., Bernardo M., Kurdziel K.A., Turkbey B., Choyke P.L. (2013). Functional and molecular imaging of localized and recurrent prostate cancer. Eur. J. Nucl. Med. Mol. Imaging.

[25] Kim C.K., Park B.K., Lee H.M. (2009). Prediction of locally recurrent prostate cancer after radiation therapy: Incremental value of 3T diffusion-weighted MRI. J. Magn. Reson. Imaging.

[26] Kim C.K., Park B.K., Park W., Kim S.S. (2010). Prostate MR imaging at 3T using a phased-arrayed coil in predicting locally recurrent prostate cancer after radiation therapy: Preliminary experience. Abdom. Imaging.

[27] Tamada T., Sone T., Jo Y., Hiratsuka J., Higaki A., Higashi H., Ito K. (2011). Locally recurrent prostate cancer after high-dose-rate brachytherapy: The value of diffusion-weighted imaging, dynamic contrast-enhanced MRI, and T2-weighted imaging in localizing tumors. AJR Am. J. Roentgenol..

[28] Radzina M., Tirane M., Roznere L., Zemniece L., Dronka L., Kalnina M., Mamis E., Biederer J., Lietuvietis V., Freimanis A. (2020). Accuracy of ^68^Ga-PSMA-11 PET/CT and multiparametric MRI for the detection of local tumor and lymph node metastases in early biochemical recurrence of prostate cancer. Am. J. Nucl. Med. Mol. Imaging.

[29] Satapathy S., Singh H., Kumar R., Mittal B.R. (2021). Diagnostic Accuracy of ^68^Ga-PSMA PET/CT for Initial Detection in Patients with Suspected Prostate Cancer: A Systematic Review and Meta-Analysis. AJR Am. J. Roentgenol..

[30] Petersen L.J., Zacho H.D. (2020). PSMA PET for primary lymph node staging of intermediate and high-risk prostate cancer: An expedited systematic review. Cancer Imaging Off. Publ. Int. Cancer Imaging Soc..

[31] Jannusch K., Bruckmann N.M., Morawitz J., Boschheidgen M., Quick H.H., Herrmann K., Fendler W.P., Umutlu L., Stuschke M., Hadaschik B. (2023). Recurrent prostate cancer: Combined role for MRI and PSMA-PET in ^68^Ga-PSMA-11 PET/MRI. Eur. Radiol..

[32] Albisinni S., Aoun F., Marcelis Q., Jungels C., Al-Hajj Obeid W., Zanaty M., Tubaro A., Roumeguere T., De Nunzio C. (2018). Innovations in imaging modalities for recurrent and metastatic prostate cancer: A systematic review. Minerva Urol. Nefrol..

[33] Rasing M., van Son M., Moerland M., de Keizer B., Wessels F., Jonges T., van de Pol S., Eppinga W., Noteboom J., Lagendijk J. (2022). Value of Targeted Biopsies and Combined PSMA PET/CT and mp-MRI Imaging in Locally Recurrent Prostate Cancer after Primary Radiotherapy. Cancers.

[34] da Silva R.D., Kim F.J. (2018). Prostate cancer—Local treatment after radiorecurrence: Salvage cryoablation. Int. Braz. J. Urol..

[35] Bahn D.K., Lee F., Badalament R., Kumar A., Greski J., Chernick M. (2002). Targeted cryoablation of the prostate: 7-year outcomes in the primary treatment of prostate cancer. Urology.

[36] Miller R.J., Cohen J.K., Shuman B., Merlotti L.A. (1996). Percutaneous, transperineal cryosurgery of the prostate as salvage therapy for post radiation recurrence of adenocarcinoma. Cancer.

[37] Leibovici D., Chiong E., Pisters L.L., Guo C.C., Ward J.F., Andino L., Prokhorova I.N., Troncoso P. (2012). Pathological characteristics of prostate cancer recurrence after radiation therapy: Implications for focal salvage therapy. J. Urol..

[38] Finley D.S., Belldegrun A.S. (2011). Salvage cryotherapy for radiation-recurrent prostate cancer: Outcomes and complications. Curr. Urol. Rep..

[39] Campbell S.P., Deivasigamani S., Arcot R., Adams E.S., Orabi H., Elshafei A., Tan W.P., Davis L., Wu Y., Chang A. (2023). Salvage Cryoablation for Recurrent Prostate Cancer Following Primary External Beam Radiotherapy or Primary Cryotherapy: A Propensity Score Matched Analysis of Mid-term Oncologic and Functional Outcomes. Clin. Genitourin. Cancer.

[40] Tan W.P., Kotamarti S., Ayala A., Mahle R., Chen E., Arcot R., Chang A., Michael Z., Seguier D., Polascik T.J. (2023). Oncological and functional outcomes for men undergoing salvage whole-gland cryoablation for radiation-resistant prostate cancer. Eur. Urol. Oncol..

[41] Spiess P.E., Levy D.A., Mouraviev V., Pisters L.L., Jones J.S. (2013). Biochemical failure predictors after prostate salvage cryotherapy. BJU Int..

[42] Wenske S., Quarrier S., Katz A.E. (2013). Salvage cryosurgery of the prostate for failure after primary radiotherapy or cryosurgery: Long-term clinical, functional, and oncologic outcomes in a large cohort at a tertiary referral centre. Eur. Urol..

[43] Lian H., Yang R., Lin T., Wang W., Zhang G., Guo H. (2016). Salvage cryotherapy with third-generation technology for locally recurrent prostate cancer after radiation therapy. Int. Urol. Nephrol..

[44] Ghafar M.A., Johnson C.W., De La Taille A., Benson M.C., Bagiella E., Fatal M., Olsson C.A., Katz A.E. (2001). Salvage cryotherapy using an argon based system for locally recurrent prostate cancer after radiation therapy: The Columbia experience. J. Urol..

[45] Ward J.F., Jones J.S. (2012). Focal cryotherapy for localized prostate cancer: A report from the National Cryo on-line database (cold) registry. BJU Int..

[46] Spiess P.E., Levy D.A., Pisters L.L., Mouraviev V., Jones J.S. (2013). Outcomes of salvage prostate cryotherapy stratified by pre-treatment PSA: Update from the COLD registry. World J. Urol..

[47] Babaian R.J., Donnelly B., Bahn D., Baust J.G., Dineen M., Ellis D., Katz A., Pisters L., Rukstalis D., Shinohara K. (2008). Best practice statement on cryosurgery for the treatment of localized prostate cancer. J. Urol..

[48] Izawa J.I., Perrotte P., Greene G.F., Scott S., Levy L., McGuire E., Madsen L., von Eschenbach A.C., Pisters L.L. (2001). Local tumor control with salvage cryotherapy for locally recurrent prostate cancer after external beam radiotherapy. J. Urol..

[49] Ismail M., Ahmed S., Kastner C., Davies J. (2007). Salvage cryotherapy for recurrent prostate cancer after radiation failure: A prospective case series of the first 100 patients. BJU Int..

[50] Li Y.H., Elshafei A., Agarwal G., Ruckle H., Powsang J., Jones J.S. (2015). Salvage focal prostate cryoablation for locally recurrent prostate cancer after radiotherapy: Initial results from the cryo on-line data registry. Prostate.

[51] Kongnyuy M., Berg C.J., Kosinski K.E., Habibian D.J., Schiff J.T., Corcoran A.T., Katz A.E. (2017). Salvage focal cryosurgery may delay use of androgen deprivation therapy in cryotherapy and radiation recurrent prostate cancer patients. Int. J. Hyperth..

[52] Tan W.P., Chang A., Sze C., Polascik T.J. (2021). Oncological and functional outcomes of patients undergoing individualized partial gland cryoablation of the prostate: A single-institution experience. J. Endourol..

[53] Tan W.P., ElShafei A., Aminsharifi A., Khalifa A.O., Polascik T.J. (2020). Salvage focal cryotherapy offers similar short-term oncologic control and improved urinary function compared with salvage whole gland cryotherapy for radiation-resistant or recurrent prostate cancer. Clin. Genitourin. Cancer.

[54] Chang X., Liu T., Zhang F., Zhao X., Ji C., Yang R., Gan W., Zhang G., Li X., Guo H. (2015). Salvage cryosurgery for locally recurrent prostate cancer after primary cryotherapy. Int. Urol. Nephrol..

[55] Bomers J.G.R., Yakar D., Overduin C.G., Sedelaar J.P.M., Vergunst H., Barentsz J.O., de Lange F., Fütterer J.J. (2013). MR imaging-guided focal cryoablation in patients with recurrent prostate cancer. Radiology.

[56] de Castro Abreu A.L., Bahn D., Leslie S., Shoji S., Silverman P., Desai M.M., Gill I.S., Ukimura O. (2013). Salvage focal and salvage total cryoablation for locally recurrent prostate cancer after primary radiation therapy. BJU Int..

[57] Kasivisvanathan V., Emberton M., Ahmed H.U. (2013). Focal therapy for prostate cancer: Rationale and treatment opportunities. Clin. Oncol..

[58] Aminsharifi A., Jibara G., Tsivian E., Tsivian M., Elshafei A., Polascik T.J. (2019). Salvage prostate cryoablation for the management of local recurrence after primary cryotherapy: A retrospective analysis of functional and intermediate-term oncological outcomes associated with a second therapeutic freeze. Clin. Genitourin. Cancer.

[59] Boissier R., Sanguedolce F., Territo A., Gaya J., Huguet J., Rodriguez-Faba O., Regis F., Gallioli A., Vedovo F., Martinez C. (2020). Partial salvage cryoablation of the prostate for local recurrent prostate cancer after primary radiotherapy: Step-by-step technique and outcomes. Urol. Video J..

[60] Perera M., Vilaseca A., Tin A.L., Nguyen D.P., Corradi R.B., Touijer A.S., Martin-Malburet A.G., Alvim R., Benfante N., Sjoberg D.D. (2022). Morbidity of salvage radical prostatectomy: Limited impact of the minimally invasive approach. World J. Urol..

[61] Pisters L.L., Leibovici D., Blute M., Zincke H., Sebo T.J., Slezak J.M., Izawa J., Ward J.F., Scott S.M., Madsen L. (2009). Locally recurrent prostate cancer after initial radiation therapy: A comparison of salvage radical prostatectomy versus cryotherapy. J. Urol..

[62] Vora A., Agarwal V., Singh P., Patel R., Rivas R., Nething J., Muruve N. (2016). Single-institution comparative study on the outcomes of salvage cryotherapy versus salvage robotic prostatectomy for radio-resistant prostate cancer. Prostate Int..

[63] Friedlander D.F., Gu X., Prasad S.M., Lipsitz S.R., Nguyen P.L., Trinh Q.-D., Sun M., Hu J.C. (2014). Population-based Comparative Effectiveness of Salvage Radical Prostatectomy vs Cryotherapy. Urology.

[64] Autran-Gomez A.M., Scarpa R.M., Chin J. (2012). High-intensity focused ultrasound and cryotherapy as salvage treatment in local radio-recurrent prostate cancer. Urol. Int..

[65] Uchida T., Illing R.O., Cathcart P.J., Emberton M. (2006). To what extent does the prostate-specific antigen nadir predict subsequent treatment failure after transrectal high-intensity focused ultrasound therapy for presumed localized adenocarcinoma of the prostate?. BJU Int..

[66] Ben Cheikh A., Girouin N., Ryon-Taponnier P., Mège-Lechevallier F., Gelet A., Chapelon J.Y., Lyonnet D., Rouvière O. (2008). Détection par IRM des récidives locales du cancer de prostate après traitement par ultrasons focalisés de haute intensité (HIFU) transrectaux: Étude préliminaire [MR detection of local prostate cancer recurrence after transrectal high-intensity focused US treatment: Preliminary results]. J. Radiol..

[67] Williams A.K., Martínez C.H., Lu C., Ng C.K., Pautler S.E., Chin J.L. (2011). Disease-free survival following salvage cryotherapy for biopsy-proven radio-recurrent prostate cancer. Eur. Urol..

[68] Choyke P.L., Bouchelouche K. (2019). Prostate specific membrane antigen (PSMA) imaging: The past is prologue. Transl. Androl. Urol..

[69] Bologna E., Ditonno F., Licari L.C., Franco A., Manfredi C., Mossack S., Pandolfo S.D., De Nunzio C., Simone G., Leonardo C. (2024). Tissue-Based Genomic Testing in Prostate Cancer: 10-Year Analysis of National Trends on the Use of Prolaris, Decipher, ProMark, and Oncotype DX. Clin. Pract..

[70] Monaco A., Sommer J., Okpara C., Lischalk J.W., Haas J., Corcoran A., Katz A. (2022). Comparative results of focal-cryoablation and stereotactic body radiotherapy in the treatment of unilateral, low-to-intermediate-risk prostate cancer. Int. Urol. Nephrol..

[71] Lewin R., Amit U., Laufer M., Berger R., Dotan Z., Domachevsky L., Davidson T., Portnoy O., Tsvang L., Ben-Ayun M. (2021). Salvage re-irradiation using stereotactic body radiation therapy for locally recurrent prostate cancer: The impact of castration sensitivity on treatment outcomes. Radiat. Oncol..

